# Genistein promotes ionizing radiation-induced cell death by reducing cytoplasmic Bcl-xL levels in non-small cell lung cancer

**DOI:** 10.1038/s41598-017-18755-3

**Published:** 2018-01-10

**Authors:** Zhimin Zhang, Feng Jin, Xiaojuan Lian, Mengxia Li, Ge Wang, Baohua Lan, Hao He, Guo-Dong Liu, Yan Wu, Guiyin Sun, Cheng-Xiong Xu, Zhen-Zhou Yang

**Affiliations:** 1 Cancer Center, Daping Hospital and Research Institute of Surgery, Third Military Medical University, Chongqing, 400042 China; 2 Department of tumor blood, Jiangjin central hospital of Chongqing, Chongqing, 400042 China; 3 Eighth Department, Daping Hospital and Research Institute of Surgery, Third Military Medical University, Chongqing, 400042 China

## Abstract

Genistein (GEN) has been previously reported to enhance the radiosensitivity of cancer cells; however, the detailed mechanisms remain unclear. Here, we report that GEN treatment inhibits the cytoplasmic distribution of Bcl-xL and increases nuclear Bcl-xL in non-small cell lung cancer (NSCLC). Interestingly, our *in vitro* data show that ionizing radiation IR treatment significantly increases IR-induced DNA damage and apoptosis in a low cytoplasmic Bcl-xL NSCLC cell line compared to that of high cytoplasmic Bcl-xL cell lines. In addition, clinical data also show that the level of cytoplasmic Bcl-xL was negatively associated with radiosensitivity in NSCLC. Furthermore, we demonstrated that GEN treatment enhanced the radiosensitivity of NSCLC cells partially due to increases in Beclin-1-mediated autophagy by promoting the dissociation of Bcl-xL and Beclin-1. Taken together, these findings suggest that GEN can significantly enhance radiosensitivity by increasing apoptosis and autophagy due to inhibition of cytoplasmic Bcl-xL distribution and the interaction of Bcl-xL and Beclin-1 in NSCLC cells, respectively.

## Introduction

Radiotherapy is an important method for malignant tumor treatment. However, radiation therapy often causes normal tissue injury, and many types of cancer show resistance to radiation therapy^1,2^. Thus, enhancing the radiosensitivity of tumor cells and protecting the remaining normal tissues are important clinical concerns in cancer radiotherapy. According to previous reports, an adjuvant drug can be used during radiotherapy to achieve a better clinical outcome, for example, genistein (GEN). GEN is the main isoflavone component in soybeans; it can significantly enhance the radiosensitivity of tumor cells^3^, and it attenuates inflammatory injuries in normal tissue caused by ionizing radiation (IR)^4^. These anti-tumor effects of GEN were identified in both *in vitro* and in clinical cases of a wide variety of cancer types, including prostate cancer, breast cancer, colon cancer, gastric cancer, lung cancer, pancreatic cancer, and lymphoma^5–8^. Studies show that GEN improves the effectiveness of either radio- or chemotherapy in cancer cells by enhancing apoptosis and autophagy^9,10^. However, the detailed mechanism by which GEN enhances the apoptosis and autophagy induced by oncotherapy in cancer remains unclear.

Autophagy is the lysosomal degradation pathway^11^, and it exerts opposing functions in response to IR-induced stress in tumor cells. One such function is cytoprotective; inhibition of this activity can sensitize cancer cells to treatment modalities. However, excessive autophagy promotes the death of tumor cells^12,13^. In lung cancer, studies show that increased autophagy dramatically abrogates radioresistance^14,15^. Apoptosis is also a desired effect of anti-tumor therapy, and the relationship between autophagy and apoptosis may depend on the biological context in which these events occur^16,17^. The dysregulation of apoptosis is a common phenomenon in cancer cells and is one mechanism by which cancer cells can resist oncotherapy. Bcl-xL is an anti-apoptotic protein, and increased expression of Bcl-xL was closely associated with radio- and chemotherapy resistance^18^. Studies show that a combination treatment of IR and a Bcl-xL inhibitor exerts a synergistic effect by activating the Bak-apoptosis pathway in cancer cells that are resistant to oncotherapy^19,20^. Bcl-xL also regulates cellular autophagy by interacting with Beclin-1 to inhibit the initiation of Beclin-1-mediated autophagy^21,22^. Studies show downregulation of Bcl-xL expression with specific siRNAs can activate autophagy and promote cancer cell death^23,24^, suggesting that Bcl-xL plays an key role in the crosstalk between autophagy and apoptosis.

Our study shows that GEN treatment inhibits cytoplasmic translocation of Bcl-xL in NSCLC cells, and the level of cytoplasmic Bcl-xL was negatively correlated with radiosensitivity in NSCLC. In addition, our data show that GEN treatment can enhance IR-induced cell death in NSCLC cells by simultaneously activating apoptosis and autophagy. Furthermore, we identified that increased autophagy by GEN is due to the promotion of Bcl-xL dissociation from Beclin-1, thereby activating Beclin-1 induced autophagy.

## Results

### GEN reduced cytoplasmic of Bcl-xL levels in NSCLC cells

**Figure 1:**
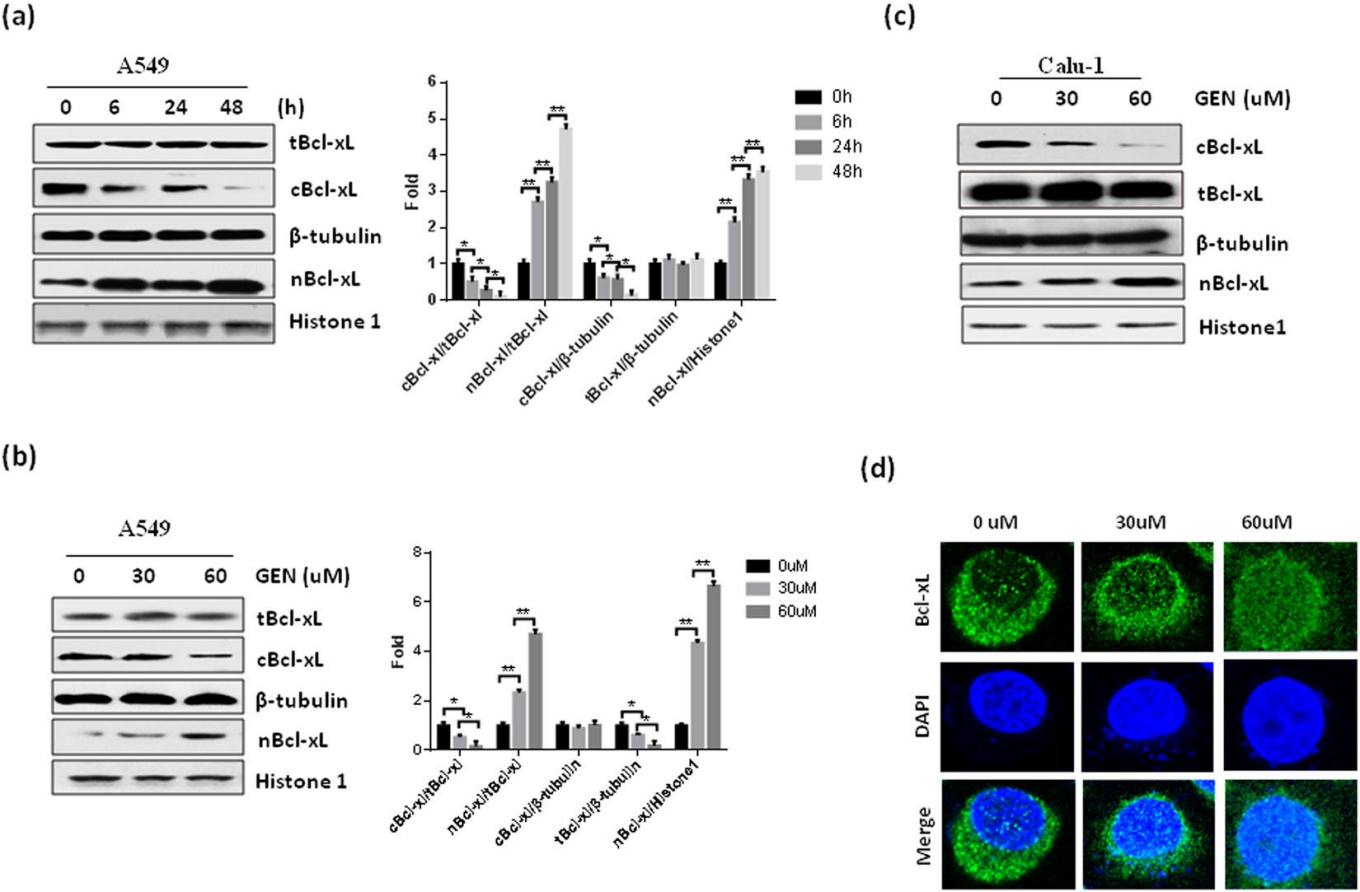
GEN affects the subcellular distribution of Bcl-xL in NSCLC cells. (**a**) A549 cells were treated with 60 µM GEN for the indicated times. Then, either nuclear and cytoplasmic or total proteins were isolated and subjected to Western blot to detect Bcl-xL. The experiment was repeated three times. (**b**) A549 cells were treated with the indicated concentration of GEN for 24 h. Then, either nuclear and cytoplasmic or total proteins were isolated and subjected to Western blot to detect Bcl-xL. The experiment was repeated three times. (**c**) Bcl-xL levels in Calu-1 cells were measured at 24 h after treatment with the indicated concentration of GEN. (**d**) Representative fluorescence images of the distribution of Bcl-xL protein in A549 cells at 24 h post-treatment with the indicated concentration of GEN. Bcl-xL expression was detected with an anti-Bcl-xL primary antibody and a FITC-labeled secondary antibody. The cell nuclei were stained with DAPI. tBcl-xL, total Bcl-xL; cBcl-xL, cytoplasmic Bcl-xL; nBcl-xL, nuclear Bcl-xL. *P < 0.05; **P < 0.01, ***P< 0.001.

Bcl-xL is an important anti-apoptotic protein. Our *in vitro* experiment shows that GEN treatment significantly reduces the levels of cytoplasmic Bcl-xL while simultaneously increasing the nuclear Bcl-xL levels in a time- and dose-dependent manner in A549 cells (Fig. 1a,b). However, GEN does not affect the total expression of Bcl-xL in A549 cells (Fig. 1a,b). These results, we confirmed in another NSCLC cell line, Calu-1. As shown in Fig. 1c, similar with A549 cells, GEN treatment significantly reduced cytoplasmic levels of Bcl-xL as well as increased nuclear Bcl-xL levels in Calu-1 cells, however, does not affect the total expression of Bcl-xL in Calu-1 cells. Finally, we used immunofluorescence analysis to confirm the effect of GEN on Bcl-xL subcellular distribution. As shown in Fig. 1d, GEN treatment significantly inhibited cytoplasm distribution of Bcl-xL while increasing nuclear Bcl-xL levels in A549 cells in a dose-dependent manner. Taken together, these results suggest that GEN affects the subcellular distribution of Bcl-xL in NSCLC cells.

### Cytoplasmic Bcl-xL levels are negatively associated with radiosensitivity in NSCLC

**Figure 2:**
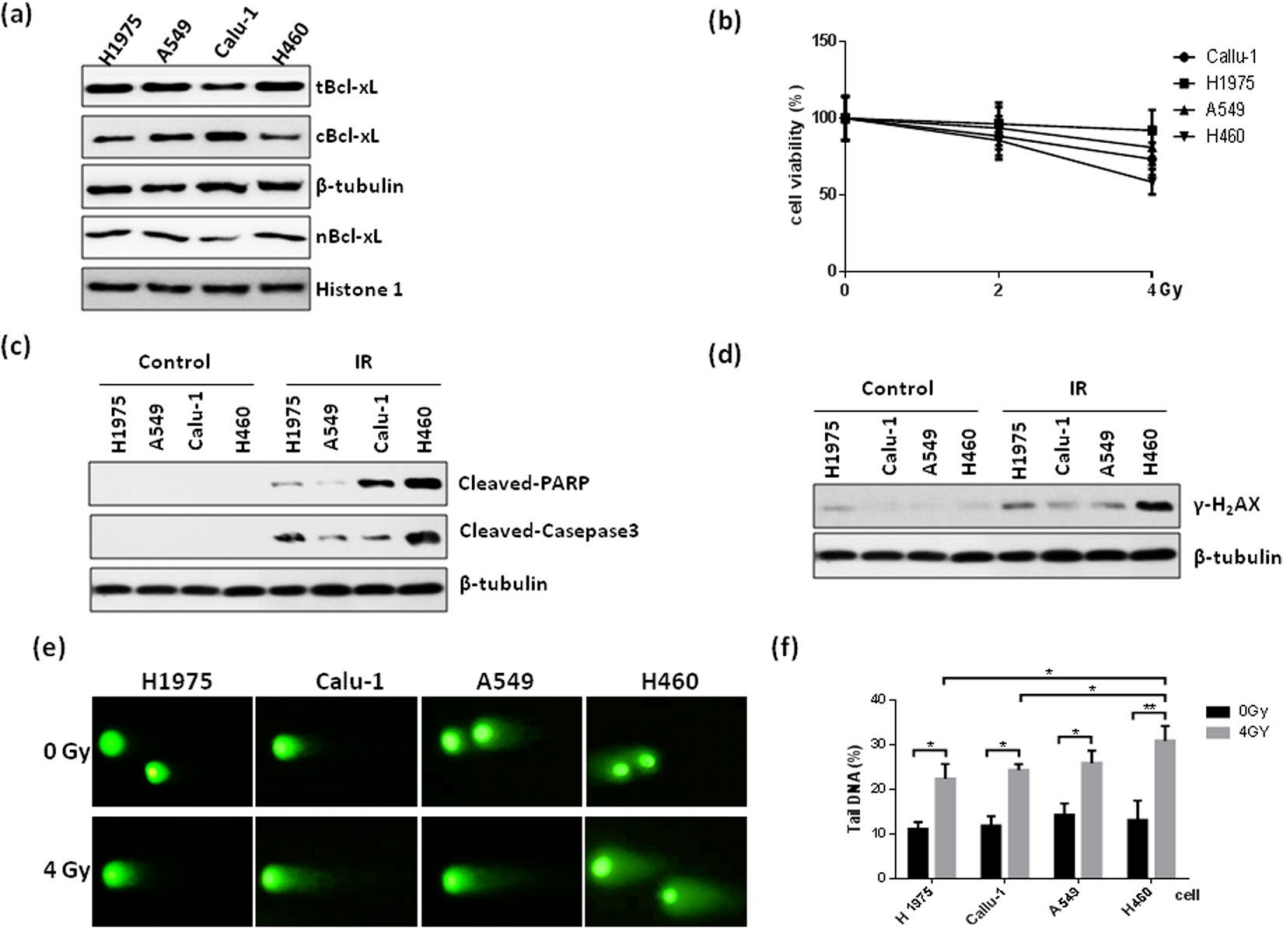
Effect of cytoplasmic Bcl-xL on DNA damage and apoptosis induced by IR in NSCLC cells. (**a**) Cytoplasmic and nuclear proteins were isolated form the indicated NSCLC cell lines and subjected to Western blot to detect Bcl-xL. (**b**) The noted cells were treated with the indicated dose of IR. After 24 h of IR treatment, cell viability was measured using CCK-8 assays. (**c**) The indicated cells were treated with 4 Gy of IR. After 2 h of IR treatment, cells were subjected to Western blot analysis of cleaved PARP and cleaved caspase-3. (**d**) γ-H2AX was measured in the indicated cells at 2 h after of treatment with 4 Gy of IR. (**e**) DNA damage was analyzed in the indicated cells by performing neutral Comet assays at 2 h after of treatment with the indicated dose of IR. (**f**) Quantification of DNA damage. *P < 0.05; **P < 0.01, ***P < 0.001.

**Figure 3:**
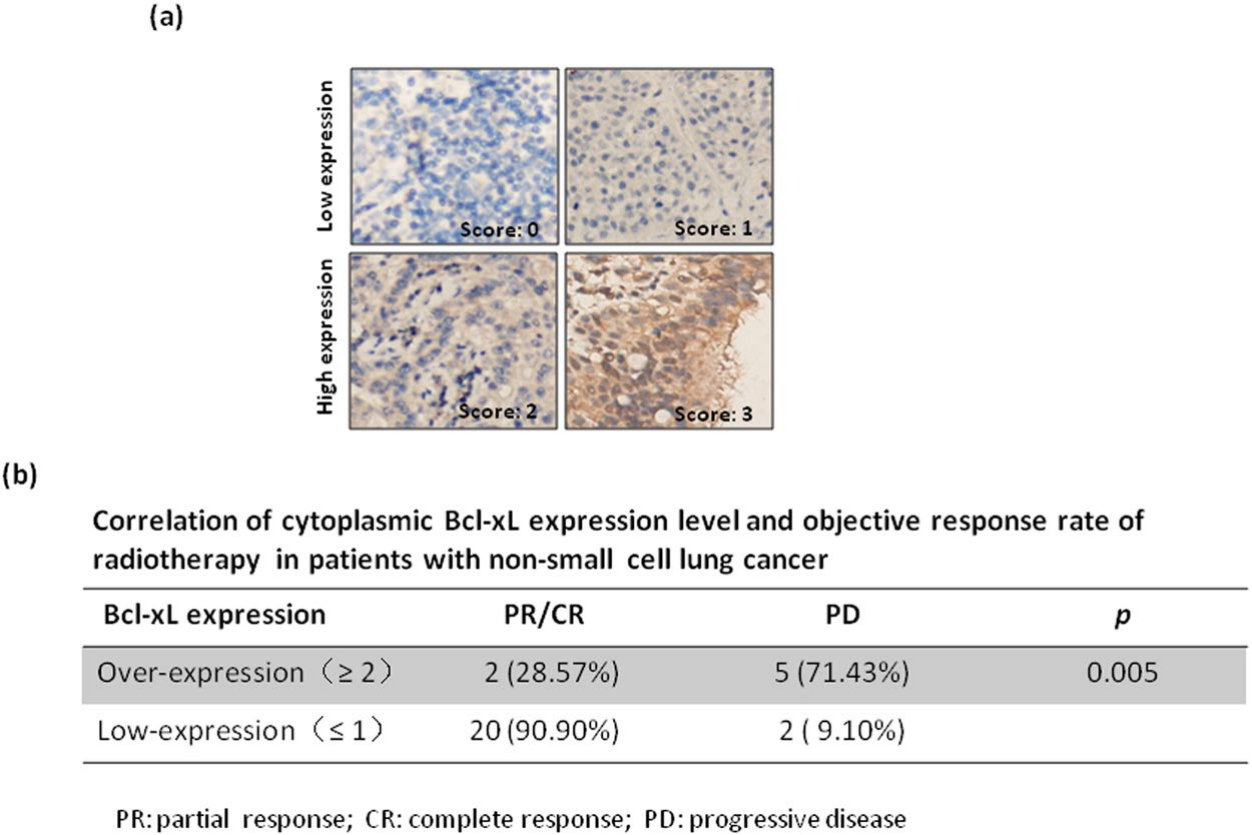
Scoring standards for Bcl-xL immunohistochemistry (IHC) staining. (**a**) Bcl-xL levels were determined by IHC in biopsy samples from patients with NSCLC. The representative images are the standard scoring images used to evaluate the intensity of Bcl-xL staining. (**b**) The correlation between cytoplasmic Bcl-xL and objective response rate of radiotherapy in patients with NSCLC was analyzed.

Next, we investigated the correlation between the levels of cytoplasmic Bcl-xL and radiosensitivity in NSCLC cell lines. First, we measured the expression level of cytoplasmic Bcl-xL in different NSCLC cell lines. As shown in Fig. 2a, H460 cells have lower cytoplasmic Bcl-xL levels and higher nuclear Bcl-xL levels compared to other NSCLC cells, including H1975, Calu-1 and A549 cells. Interestingly, the CCK-8 assay shows that H460 cells are more sensitive to IR treatment compared to the NSCLC cell lines that have higher basal cytoplasmic Bcl-xL levels (Fig. 2b). Consistent with this result, treatment with IR significantly induced more expression of the pro-apoptotic protein cleaved PARP, cleaved caspase-3 (Fig. 2c) and DNA damage (Fig. 2d–f) in H460 cells compared to the other NSCLC cell lines. Furthermore, we investigated the impact of cytoplasmic Bcl-xL levels on the objective response rate (ORR) of radiotherapy in NSCLC patents. The expression of Bcl-xL was analyzed in tumor samples from 29 NSCLC patients using immunohistochemistry and categorized into two groups based the immunostaining score (Fig. 3a). As shown in Fig. 3b, 22 and 7 cases of the 29 tested NSCLC samples showed low and high expression of cytoplasmic Bcl-xL, respectively. Interestingly, in the cytoplasmic Bcl-xL high expression group, only 28.57% of NSCLC patients responded to radiotherapy, whereas the remaining 75.9% of patients experienced either recurrence or new metastasis (Fig. 3b). In contrast, 90.9% of NSCLC patients in the cytoplasmic Bcl-xL low expression group responded to radiotherapy, whereas the remaining 9.1% patients did not respond (Fig. 3b). These findings suggest that level of cytoplasmic Bcl-xL was negatively associated with radiosensitivity in NSCLC.

### GEN enhances the radiosensitivity of NSCLC cells by enhancing IR-induced DNA damage and apoptosis

**Figure 4:**
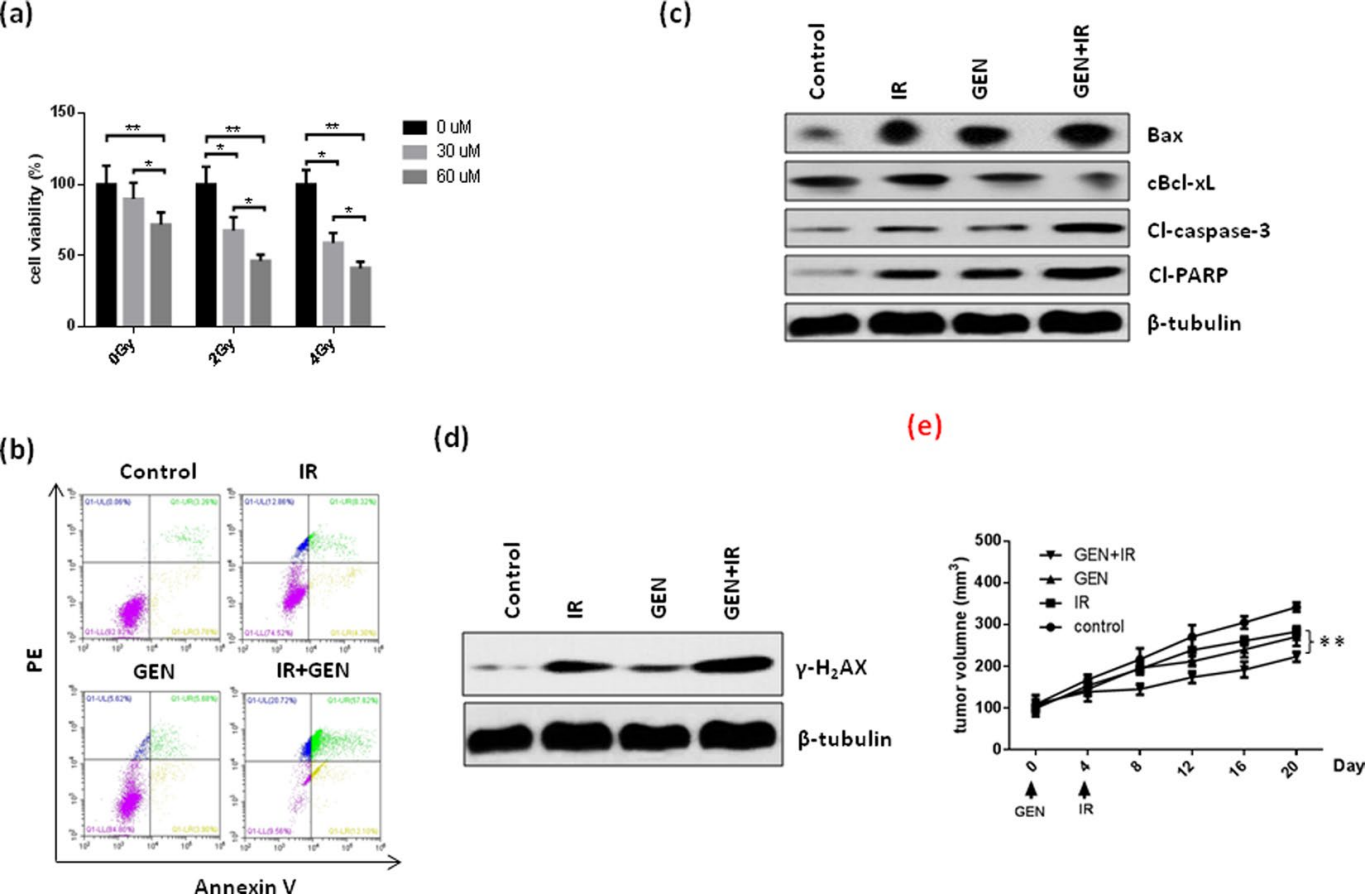
GEN enhances DNA damage and apoptosis induced by IR in A549 cells. (**a**) A549 cells were treated with the indicated concentration of GEN for 24 h followed by treatment with the indicated dose of IR. Cell viability was evaluated using the CCK-8 assay after 24 h of IR treatment. (**b**) After treatment with 60 µM GEN for 24 h, A549 cells were either untreated or administered 4 Gy of IR. After 24 h of IR treatment, the cells were subjected to flow cytometry analysis. (**c**,**d**) A549 cells were either untreated or pretreated with 60 µM GEN for 24 h. Then, the subsequent cells were either untreated or administered 4 Gy of IR. After 2 h of IR treatment, the cells were harvested and subjected to Western blot analysis. (**e**) Combination treatment of GEN and IR significantly inhibited tumor growth compared to single treatment in A549 xenograft model. *P < 0.05; **P < 0.01, ***P < 0.001.

We next investigated whether the combination of IR and GEN could exert a synergistic effect on inhibition of NSCLC cell viability. As shown in Fig. 4a and b, the combination of GEN and IR treatment significantly inhibited more cell growth and induced more apoptosis compared to either GEN or IR treatment alone in A549 cells. Consistent with these results, the combination treatment of GEN and IR significantly increased the expression of the DNA damage marker protein γ-H2AX, the pro-apoptotic protein Bax, cleaved-PARP and -caspase-3 in A549 cells compared to cells receiving solo treatments of either GEN or IR (Fig. 4c and d). Additionally, we detected significantly reduced levels of cytoplasmic Bcl-xL in the GEN and IR combination treatment group (Fig. 4d). Furthermore, we confirmed that synergistic inhibition effect of combination of IR and GEN treatment on NSCLC growth using A549 xenograft model. Consistent with *in vitro* cell viability assay, the combination treatment of GEN and IR significantly inhibited tumor growth in xenogaft model (Fig. 4e). Taken together, these findings suggest that GEN can enhance the radiosensitivity of NSCLC cells through reducing plasmic Bcl-xL levels and promoting IR-induced DNA damage and apoptosis.

### GEN enhances the radiosensitivity of NSCLC cells through promote autophagy

**Figure 5:**
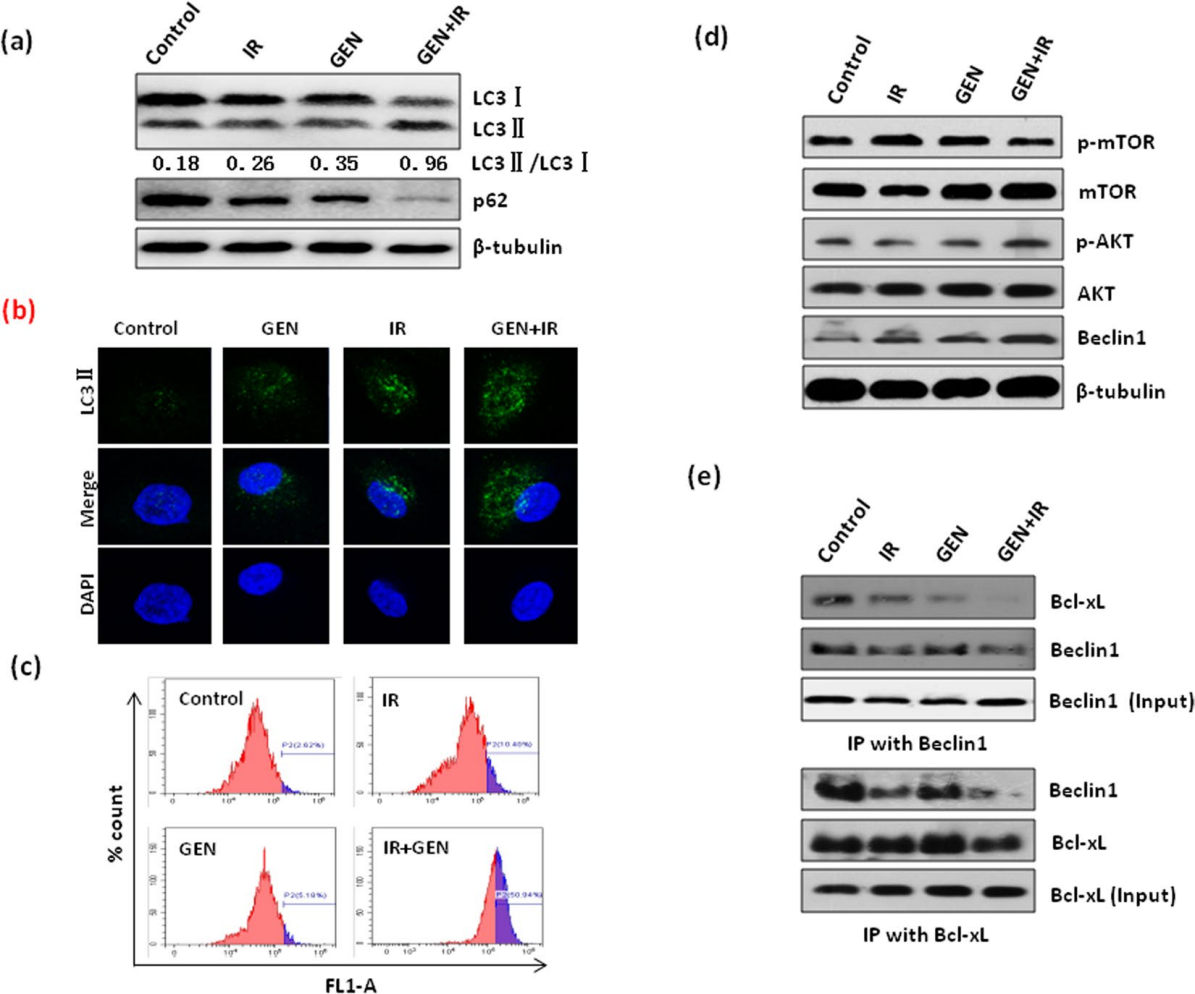
Role of Bcl-xL/Beclin-1 in combination with GEN in IR-induced autophagy. A549 cells were in the presence or absence of 60 µM GEN for 24 h. Then, cells were either untreated or administered 4 Gy of IR. (**a**) After 2 h of IR treatment, cells were subjected to Western blot to detect autophagy-related proteins. (**b**) After 24 h of IR treatment, cells were subjected to immunofluororescense staining of LC3. Green, LC3; Blue, DAPI. (**c**) After 24 h of IR treatment, the cells were subjected to flow cytometry for autophagy detection. (**d**) After 2 h of IR treatment, the expression levels of mTOR, p-mTOR, β-tubulin, p-Akt, Akt, and Beclin-1 were measured by Western blot. (**e**) After 2 h of IR treatment, the cells were subjected to immunoprecipitation.

Finally, we investigated whether GEN affects IR-induced autophagy in NSCLC cells because previous studies have shown that GEN can affect chemotherapy- induced autophagy^10^. As shown Fig. 5a, the combined treatment of GEN and IR significantly increased the level of autophagy maker protein LC3II while decreasing p62 compared to either control cells or cells receiving a single treatment. Consistent with this Western blot data, IF staining show that LC3 expression was significantly increased in GEN and IR combined treatment group compare to either control or single treatment (Fig. 5b), suggesting that the combination of GEN and IR treatment exerts synergistic effects on autophagy. This phenomenon was further confirmed by flow cytometry (Fig. 5c). Because the AKT/mTOR pathway is involved in Beclin-1 induced autophagy, we investigated the effects of GEN and IR combination on the AKT/mTOR/Beclin-1 axis in NSCLC cells. Our results show that only Beclin-1 expression was significantly increased after treatment with the GEN and IR combination compared to the control and single treatment groups (Fig. 5d). Next, we investigated the effects of GEN and IR combination on Bcl-xL/Beclin-1 binding because studies show that Bcl-xL inhibits Beclin-1-mediated autophagy by binding to Beclin-1 to inhibit autophagosome formation^21,25^. As shown in Fig. 5e, the combination of GEN and IR significantly promotes the dissociation of Bcl-xL/Beclin-1 in A549 cells. Together, these data suggest that GEN can enhance IR-induced autophagy by promoting the dissociation of Bcl-xL and Beclin-1.

**Figure 6:**
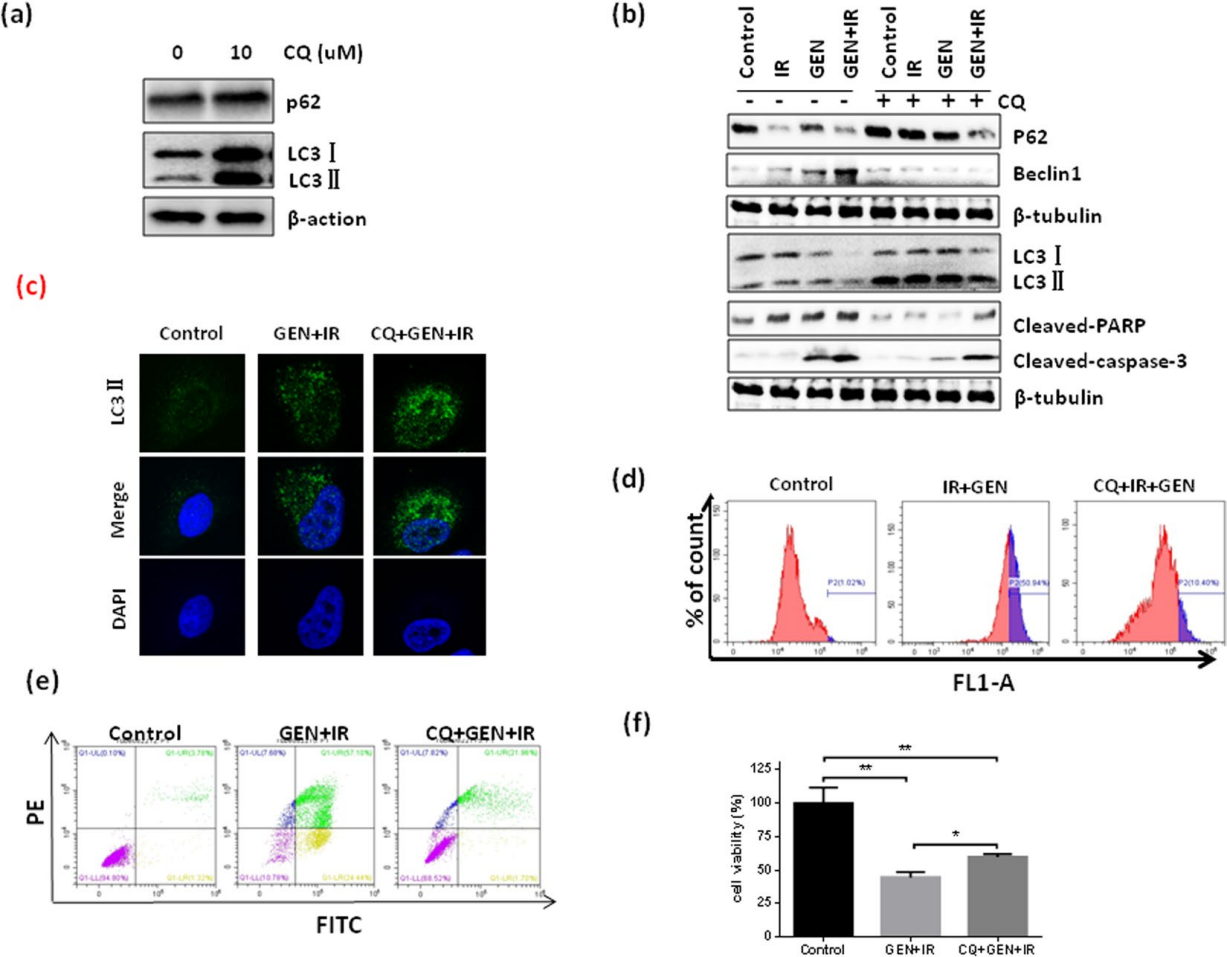
Role of autophagy in apoptosis induction. (**a**) The expression levels of p62 and LC3 proteins in A549 cells were measured after 12 h of 10 µM hydroxychloroquine (CQ) treatment. (**b**) A549 cells either in the presence or absence of 10 µM CQ for 4 h were treated with or without 60 µM GEN. After 24 h of GEN treatment, cells were treated with 0 or 4 Gy of IR. After 2 h of IR treatment, cells were subjected to Western blot. To measure (**c**) LC3 expression, (**d**) autophagy, (**e**) apoptosis and (**f**) cell viability, A549 cells were either untreated or subjected to 10 µM CQ for 4 h, after which the cells were either in the presence or absence of 60 µM GEN. After 24 h of GEN treatment, the cells were treated with 2 Gy of IR. After 24 h of IR treatment, cells were then subjected to flow cytometry analysis. *P < 0.05; **P < 0.01, ***P < 0.001.

Then, we investigated whether autophagy plays a critical role on the cell death induced by the combination of IR and GEN. Here, we used hydroxychloroquine (CQ) as an autophagy inhibitor (Fig. 6a). Our data show that inhibition of autophagy by CQ abolished IR and GEN combination treatment induced high expression of cleaved PARP and cleaved caspase-3 in A549 cells (Fig. 6b). In addition, the flow cytometry results show that the inhibition of autophagy dramatically reduced apoptosis induced by the GEN and IR combination treatment (Fig. 6c and d). Those data indicate that the combined GEN and IR treatment-induced apoptosis is partially due to the stimulation of autophagy in NSCLC cells.

## Discussion

Radiotherapy remains one of the prime treatment modalities for many cancers. However, the clinical concern is the subset of cancer patients who show resistance to radiotherapy. GEN is an isoflavone isolated from soy, and previous reports have highlighted that GEN can enhance the efficacy of radiotherapy in numerous tumor types. Consistent with previous reports, we also demonstrated the significantly enhanced efficacy of radiotherapy when combined with GEN in NSCLC cells. More importantly, for the first time, we demonstrated that GEN enhanced the radiosensitivity of NSCLC cells by simultaneously stimulating apoptosis and autophagy. Interestingly, studies show that GEN not only promotes the therapeutic efficacy of radiation in lung cancer but also protects normal lung tissues from radiation^26^. Together, these findings suggest that GEN treatment may be a useful strategy to enhance radiotherapy efficacy and protect normal tissues from IR in NSCLC patients.

In the current study, we also clarified the radiosensitivity regulatory mechanism of GEN in NSCLC cells. Bcl-xL is a major anti-apoptotic protein. Studies show that IR treatment induces Bcl-xL expression, thereby causing radioresistance of NSCLC cells^27^. In addition, inhibiting the Bcl-xL pathway could improve resistance to radiotherapy in lung cancer patients^27^, suggesting that Bcl-xL plays an important role in the development of radioresistance. Previous results show that GEN treatment suppresses Bcl-xL expression to induce apoptosis in hepatoma cells^28^. Contrary to previous reports, we did not observe changes in the total expression of Bcl-xL after GEN treatment in NSCLC cells. However, our Western blot and IF analyses clearly show that GEN treatment reduces cytoplasmic levels Bcl-xL as well as increases nuclear Bcl-xL levels in NSCLC cells. In addition, our *in vitro* data show that the level of cytoplasmic Bcl-xL was negatively associated with IR-induced DNA damage and apoptosis. Consistent with the *in vitro* experiments, our clinical data revealed a significant correlation between the low cytoplasmic Bcl-xL levels and the ORR to radiotherapy. According to Subramanian *et al*., Bcl-xL interacts with pro-apoptotic proteins, including Bim and Bid, in the cytoplasm^29^, suggesting that cytoplasmic Bcl-xL plays a crucial role in apoptosis. Nuclear Bcl-xL also plays an important role in DNA damage. Nuclear Bcl-xL can inhibit the DNA damage repair gene APE1 by interacting with APE1, thus enhancing oncotherapy-induced DNA damage and apoptosis^30^. This suggests that not only total Bcl-xL levels but the distribution of Bcl-xL are also an important factor in oncotherapy-induced apoptosis. Together, these findings suggest that GEN enhances the radiosensitivity of NSCLC cells through stimulating apoptosis due to the subcellular distribution of Bcl-xL. However, the mechanism of how GEN regulates the cellular distribution of Bcl-xL is unclear and requires further research.

Autophagy is the process of self-digestion as it relates to both cell survival and cell death and plays an important role in the regulation of cell radiosensitivity. Our results show that GEN treatment promotes both autophagy and apoptosis in NSCLC cells and that the autophagy stimulates apoptosis. Cytoplasmic Bcl-xL plays a crucial role in maintaining homeostasis of apoptosis and autophagy during stress^21^. Studies show that Bcl-xL can binding to Beclin-1 and inhibit Beclin-1 mediated autophagy^21,31^. Interestingly, studies show that increased autophagy by overexpression of Beclin-1 can significantly abrogate the radioresistance of lung cancer cells^14^, suggesting that Beclin-1-mediated autophagy plays an important role in the radioresistance of lung cancer cells. Here, our data clearly show that GEN treatment can increase autophagy, and inhibiting autophagy can partially abrogate the cell death induced by the combined IR and GEN treatment in NSCLC cells. In addition, our data show that GEN treatment promotes the dissociation of Bcl-xL and Beclin-1 in NSCLC cells. Together these findings suggest that GEN treatment can enhance radiosensitivity partially by promoting Bcl-xL and Beclin-1 dissociation to stimulate autophagy in NSCLC cells. However, the mechanism of how GEN affect the interaction of Bcl-xL and Beclin-1 is still unclear.

In conclusion, GEN inhibits the cytoplasmic distribution of Bcl-xL, and the reduced levels of cytoplasmic Bcl-xL are closely associated with the radiosensitivity of NSCLC cells. GEN treatment also enhanced radiosensitivity by increasing DNA damage induced apoptosis and Beclin-1-mediated autophagy due to the promoted dissociation of Bcl-xL and Beclin-1. Our findings suggesting that GEN could be a potent therapeutic agent to enhance the sensitivity of radiotherapy in resistant NSCLC.

## Materials and Methods

### Materials

Dulbecco’s modified Eagle’s medium (DMEM) and fetal bovine serum (FBS) were obtained from Invitrogen (Grand Island, NY, USA). Genistein (GEN), hydroxychloroquine, penicillin, streptomycin and dimethyl sulfoxide (DMSO) were purchased from Sigma-Aldrich (St. Louis, MO, USA). Immunoprecipitation (IP) lysis buffer, Dynabeads® Protein G, fluorescence isothiocyanate (FITC)-Annexin V and PI were purchased from Life Technologies Corporation (Carlsbad, CA, USA). Antibody against Bcl-xL was purchased from Santa Cruz Biotechnology (Santa Cruz, CA, USA). Antibodies against mTOR, p-mTOR, p62, Bax, β-tubulin, p-Akt, Akt, PARP, horseradish peroxidase (HRP)-conjugated anti-mouse and anti-rabbit IgG antibodies were from Abcam (Cambridge, MA, USA). Beclin1, LC3, γ-H2AX, and caspase-3 were purchased from Cell Signaling Technology (Danvers, MA, USA). Cyto-ID Autophagy Detection Kits were purchased from Enzo Life Sciences (Farmingdale, NY, USA), and NE-PER(R) Nuclear and Cytoplasmic Extraction Kits were from Thermo Scientific Pierce (Rockford, IL, USA).

### Cell culture

Human NSCLC cell line A549, Calu-1, H1975 and H460 were obtained from the American Type Culture Collection (ATCC; Manassas, VA, USA). All cells were cultured in MEM medium containing 10% FBS and 50 mg/mL penicillin/streptomycin, in a 5% CO2, 37 °C humidified incubator.

### Western blot analysis

Equal amounts of proteins were separated on SDS-polyacrylamide gels, and transferred to PVDF membranes, then blocked with 5% nonfat dry milk in TBST for 1 h at room temperature. Next, membranes were incubated with primary antibody for overnight at 4 °C, followed by 1 h incubation at room temperature with horseradish peroxidase-conjugated secondary antibodies. Finally reacted with chemiluminescent staining reagents. The stained protein bands were visualized with BioMax-Light film (Eastman Kodak Co., Rochester, NY, USA), and the staining intensities of the various protein bands were obtained using Gel Doc 2000 apparatus and software (Quantity One, Bio-Rad; Hercules, CA, USA).

### Co-immunoprecipitation (Co-IP) assay

Cells were harvested by scraping, and washed once with ice-cold phosphate-buffered saline (PBS) solution; after which, cells were incubated in IP lysis buffer (Life Technologies) supplemented with a protease inhibitor cocktail (Pierce). Protein concentrations were determined using the Bradford assay (Bio-Rad, Hercules, CA, USA). A 50 μL (1.5 mg) aliquot of Dynabeads® was transferred to a tube, which was then placed on a magnet to separate the beads from the solution, and the supernatant was removed. Next, Bcl-xl or Beclin-1 antibody in 200 μL of PBS containing Tween®-20 was added to the tube, which was then incubated with rotation for 2 h at 4 °C. After removing the supernatant, an equal volume of protein extract was added to the tube, which was then incubated with rotation overnight at 4 °C. Next, the Dynabeads®-Bcl-xL-protein or Dynabeads®-Belin1-protein complex was washed 3 times with 200 μL of washing buffer, the supernatant was removed, and 20 μL of elution buffer, 10 μL of premixed NuPAGE® LDS sample buffer, and NuPAGE® sample reducing agent were added to the tube, which was then heated at 70 °C for 10 min. Finally, the tube was placed on a magnet and the sample was loaded onto a gel.

### CCK-8 assay

Cells were seeded in 96-well plates at density 2 × 10^3^/well. After 12 hrs of transfection, cells were treated as indicated. Cell viability was detected using CCK-8 kit according to manufacture’s intruction. The absorbance of each well was determined at 450 nm using a Microplate Reader 550 (Bio-Rad Laboratories).

### Comet assay

A549 cells, H1975 cells, Calu-1 and H460 cells were rinsed twice with ice-cold PBS and harvested. The cells were then re-suspended, and each suspension was exposed to IR (2 GY). Either immediately after treatment or after 2-hour post-treatment recovery incubation at 37 °C, each cell suspension was placed on ice, or an alkaline comet assay was performed using a Comet assay kit (Trevigen; Gaithersburg, MD, USA) according to the manufacturer’s instructions with modifications.

### Flow cytometry analysis

A549 cells were cultured in six-well plates and treated as indicated. Then, cells were harvested and resuspended in PBS, stained using an annexin V/propidium iodide (PI) kit (Life Technologies) following the manufacturer’s instructions, and analyzed by flow cytometry.

### Immunofluorescence (IF) and Immunohistochemistry (IHC) analysis

For IF analysis, A549 cells were treated with indicated concentrations of GEN. After 6 h of GEN treatment, cells were fixed with 3.7% paraformaldehyde in PBS for 30 min, permeabilized with 0.2% Triton X-100 in PBS for 10 min, blocked with 5% BSA in PBS for 30 min, and incubated with anti-Bcl-xl antibody overnight at 4 °C. After incubation, the cells were flooded with Texas-Red-labeled secondary antibody for 60 min, then stained with DAPI4 (6-diamidino-2-phenyl-indole dihydrochloride). Fluorescent images were observed using a laser-scanning confocal microscope (Olympus FV500, Chongqing, China) equipped with appropriate filters.

The expression of cytoplasmic Bcl-xl in human specimens were measured by IHC. After deparaffinization and blocking, slides were incubated Bcl-xl monoclonal antibody (1:200 dilution)for overnight at 4 °C; incubated with 1:50 dilution of goat anti-mouse secondary antibody for 1 h at room temperature. Finally, slides were incubated with 3,30-diaminobenzidine (DAB) substrate. Scoring for cytoplasmic Bcl-xl staining was performed as described previously by three professional pathologist^32^.

### Patients and clinical specimens

The present study enrolled cancer patients who were treated at the Daping Hospital of Third Military Medical University, China between 2011 and 2015. This study was carried out after approval by the Ethics Committee of the Daping Hospital and Research Institute of Surgery, Third Military Medical University and obtaining informed consent from all subjects. The methods in treating tissues were carried out strictly in accordance with institutional policies and approved guidelines of experiment operations.

### Animal experiments

For the subcutaneous tumor growth assay, 2 × 10^6^ A549 cells in 0.1 mL of phosphate-buffered saline (PBS) were subcutaneously injected into 6-weeks old female nude mice (5 mice per group). When tumors reached a size of approximately 100 mm^3^, mice were randomized into the following four treatment groups and started to genisetin treatment: (**a**) control; (**b**) genestein only; (**c**) radiation only; (**d**) combination of genestin and IR. Genistein was injected to mice every day by I.P. injection (100 mg/Kg body weight)^4^, until the end of animal experiment. After 3 days of genistein treatment, tumors were irradiated with 6 Gy radiation. After 20 dyas of genistein treatment, mice were sacrificed. The tumor size were measured using caliper every 4 days. Animal studies were conducted according to humane animal care standards and were approved by the Ethics of Committee of Third Military Medical University, China.

### Statistical analysis

Results are represented by mean ± S.D. Statistical significance was tested by one-way ANOVA, with *p*-value of less than 0.05 considered statistically significant.

## References

[1] Day RM (2013). Enhanced hematopoietic protection from radiation by the combination of genistein and captopril. International immunopharmacology.

[2] Kim KW, Moretti L, Mitchell LR, Jung DK, Lu B (2009). Combined Bcl-2/mammalian target of rapamycin inhibition leads to enhanced radiosensitization via induction of apoptosis and autophagy in non-small cell lung tumor xenograft model. Clinical cancer research: an official journal of the American Association for Cancer Research.

[3] Robertson KA (2001). Altered expression of Ape1/ref-1 in germ cell tumors and overexpression in NT2 cells confers resistance to bleomycin and radiation. Cancer research.

[4] Liu GD (2014). Genistein alleviates radiation-induced pneumonitis by depressing Ape1/Ref-1 expression to down-regulate inflammatory cytokines. Cell biochemistry and biophysics.

[5] Li Y, Upadhyay S, Bhuiyan M, Sarkar FH (1999). Induction of apoptosis in breast cancer cells MDA-MB-231 by genistein. Oncogene.

[6] Baxa DM, Yoshimura FK (2003). Genistein reduces NF-kappa B in T lymphoma cells via a caspase-mediated cleavage of I kappa B alpha. Biochemical pharmacology.

[7] Yu Z, Li W, Liu F (2004). Inhibition of proliferation and induction of apoptosis by genistein in colon cancer HT-29 cells. Cancer letters.

[8] Li Y, Sarkar FH (2002). Down-regulation of invasion and angiogenesis-related genes identified by cDNA microarray analysis of PC3 prostate cancer cells treated with genistein. Cancer letters.

[9] Gossner G (2007). Genistein-induced apoptosis and autophagocytosis in ovarian cancer cells. Gynecologic oncology.

[10] Suzuki R (2014). Genistein Potentiates the Antitumor Effect of 5-Fluorouracil by Inducing Apoptosis and Autophagy in Human Pancreatic Cancer Cells. Anticancer research.

[11] Levine B, Kroemer G (2008). Autophagy in the pathogenesis of disease. Cell.

[12] Yang Y (2015). Autophagy and its function in radiosensitivity. Tumour biology: the journal of the International Society for Oncodevelopmental Biology and Medicine.

[13] Gewirtz DA (2014). The four faces of autophagy: implications for cancer therapy. Cancer research.

[14] Chang SH (2012). Beclin1-induced autophagy abrogates radioresistance of lung cancer cells by suppressing osteopontin. Journal of radiation research.

[15] Shin JY (2012). Aerosol delivery of beclin1 enhanced the anti-tumor effect of radiation in the lungs of K-rasLA1 mice. Journal of radiation research.

[16] Levine B (2007). Cell biology: autophagy and cancer. Nature.

[17] Kondo Y, Kanzawa T, Sawaya R, Kondo S (2005). The role of autophagy in cancer development and response to therapy. Nature reviews. Cancer.

[18] Datta R (1995). Overexpression of Bcl-XL by cytotoxic drug exposure confers resistance to ionizing radiation-induced internucleosomal DNA fragmentation. Cell growth & differentiation: the molecular biology journal of the American Association for Cancer Research.

[19] Wu, H. *et al*. Ionizing Radiation Sensitizes Breast Cancer Cells to Bcl-2 Inhibitor, ABT-737, through Regulating Mcl-1. *Radiation research*10.1667/RR13856.1 (2014). 10.1667/RR13856.1 PMC5523983 25409124

[20] Tagscherer KE, Fassl A, Sinkovic T, Combs SE, Roth W (2012). p53-dependent regulation of Mcl-1 contributes to synergistic cell death by ionizing radiation and the Bcl-2/Bcl-XL inhibitor ABT-737. Apoptosis: an international journal on programmed cell death.

[21] Pattingre S (2005). Bcl-2 antiapoptotic proteins inhibit Beclin 1-dependent autophagy. Cell.

[22] Shin JY, Hong SH, Kang B, Minai-Tehrani A, Cho MH (2013). Overexpression of beclin1 induced autophagy and apoptosis in lungs of K-rasLA1 mice. Lung cancer.

[23] Du P (2013). Blocking Bcl-2 leads to autophagy activation and cell death of the HEPG2 liver cancer cell line. Asian Pacific journal of cancer prevention: APJCP.

[24] Du Y, Ji X (2014). Bcl-2 down-regulation by small interfering RNA induces Beclin1-dependent autophagy in human SGC-7901 cells. Cell biology international.

[25] Duan ZL, Peng ZL, Wang ZH, Yan NH (2007). [Correlation of autophagy gene Beclin1 to tumorigenesis and development of epithelial ovarian cancer]. Ai zheng=Aizheng=Chinese journal of cancer.

[26] Calveley VL (2010). Genistein Can Mitigate the Effect of Radiation on Rat Lung Tissue. Radiation research.

[27] You S (2014). Disruption of STAT3 by niclosamide reverses radioresistance of human lung cancer. Molecular cancer therapeutics.

[28] Su SJ, Chow NH, Kung ML, Hung TC, Chang KL (2003). Effects of soy isoflavones on apoptosis induction and G2-M arrest in human hepatoma cells involvement of caspase-3 activation, Bcl-2 and Bcl-XL downregulation, and Cdc2 kinase activity. Nutrition and cancer.

[29] Subramanian T, Vijayalingam S, Kuppuswamy M, Chinnadurai G (2015). Interaction of cellular proteins with BCL-xL targeted to cytoplasmic inclusion bodies in adenovirus infected cells. Virology.

[30] Ren T (2015). Small-molecule BH3 mimetic and pan-Bcl-2 inhibitor AT-101 enhances the antitumor efficacy of cisplatin through inhibition of APE1 repair and redox activity in non-small-cell lung cancer. Drug design, development and therapy.

[31] Zalckvar E, Berissi H, Eisenstein M, Kimchi A (2009). Phosphorylation of Beclin 1 by DAP-kinase promotes autophagy by weakening its interactions with Bcl-2 and Bcl-XL. Autophagy.

[32] Wang D, Luo M, Kelley MR (2004). Human apurinic endonuclease 1 (APE11) expression and prognostic significance in osteosarcoma: enhanced sensitivity of osteosarcoma to DNA damaging agents using silencing RNA APE1 expression inhibition. Molecular cancer therapeutics.

